# Systems Biology Approaches to Understand the Host–Microbiome Interactions in Neurodegenerative Diseases

**DOI:** 10.3389/fnins.2020.00716

**Published:** 2020-07-08

**Authors:** Dorines Rosario, Jan Boren, Mathias Uhlen, Gordon Proctor, Dag Aarsland, Adil Mardinoglu, Saeed Shoaie

**Affiliations:** ^1^Centre for Host-Microbiome Interactions, Faculty of Dentistry, Oral & Craniofacial Sciences, King’s College London, London, United Kingdom; ^2^Department of Molecular and Clinical Medicine, Sahlgrenska University Hospital, University of Gothenburg, Gothenburg, Sweden; ^3^Science for Life Laboratory, KTH – Royal Institute of Technology, Stockholm, Sweden; ^4^Institute of Psychiatry, Psychology and Neuroscience, King’s College London, London, United Kingdom

**Keywords:** systems biology, microbiota-gut-brain axis, neurodegenerative diseases, Parkinson’s disease, Alzheimer’s disease, biologic network, biomarker discovery, dietary therapy

## Abstract

Neurodegenerative diseases (NDDs) comprise a broad range of progressive neurological disorders with multifactorial etiology contributing to disease pathophysiology. Evidence of the microbiome involvement in the gut-brain axis urges the interest in understanding metabolic interactions between the microbiota and host physiology in NDDs. Systems Biology offers a holistic integrative approach to study the interplay between the different biologic systems as part of a whole, and may elucidate the host–microbiome interactions in NDDs. We reviewed direct and indirect pathways through which the microbiota can modulate the bidirectional communication of the gut-brain axis, and explored the evidence of microbial dysbiosis in Alzheimer’s and Parkinson’s diseases. As the gut microbiota being strongly affected by diet, the potential approaches to targeting the human microbiota through diet for the stimulation of neuroprotective microbial-metabolites secretion were described. We explored the potential of Genome-scale metabolic models (GEMs) to infer microbe-microbe and host-microbe interactions and to identify the microbiome contribution to disease development or prevention. Finally, a systemic approach based on GEMs and ‘omics integration, that would allow the design of sustainable personalized anti-inflammatory diets in NDDs prevention, through the modulation of gut microbiota was described.

## Introduction

**FIGURE 1:**
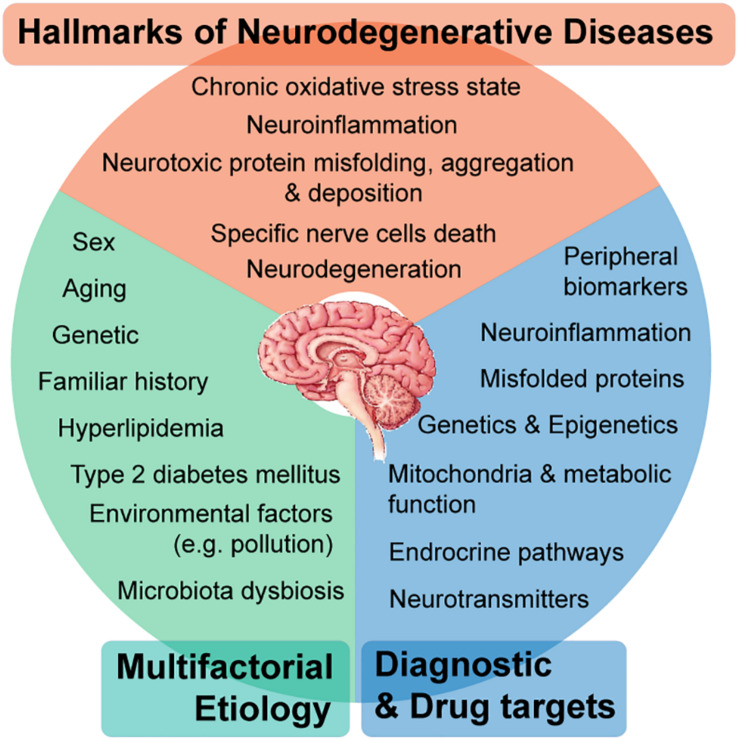
Neurodegenerative diseases (NDDs) are a broad range of complex neurological disorders, however, presenting shared hallmarks. The pathophysiology of NDDs comprises multifactorial etiologies, where genetic, environmental, and behavioral factors contribute with causal roles. Even though being distinctly manifested (e.g., compromised cognition in Alzheimer’s disease or motor functions in Parkinson’s disease), at the molecular level, there are many shared perturbed mechanisms comprising hallmarks of NDDs, which in turn leads to several potential target approaches for earlier diagnosis and effective therapeutics from a personalized medicine perspective.

Neurodegenerative diseases (NDDs) encompass a wide-range of progressive neurological disorders (Chin and Vora, 2014; Tsuiji and Yamanaka, 2014; Ma, 2018) commonly characterized by the death of specific nerve cells (Tsuiji and Yamanaka, 2014), which can lead to the overlap of clinical symptoms compromising cognitive and/or motor functions across different diseases (Ma, 2018). The World Health Organization estimates that by 2040 (Gammon, 2014), NDDs will become the second leading cause of death worldwide, taking over cancer and ranking just after cardiovascular diseases (CVD) (Gammon, 2014). Alzheimer’s disease (AD), Parkinson’s disease (PD), Frontotemporal lobar degeneration (FTLD) (Tsuiji and Yamanaka, 2014; Wang et al., 2015; Friedland and Chapman, 2017), Huntington’s disease (HD) (Tsuiji and Yamanaka, 2014; Wang et al., 2015), Multiple sclerosis (MS) and Amyotrophic lateral sclerosis (ALS) (Tsuiji and Yamanaka, 2014; Friedland and Chapman, 2017) are representative NDDs characterized by the deposition of aggregates of misfolded disease-specific neurotoxic proteins (Figure 1). This deposition is confined to specific anatomic brain regions, therefore leading to particular disease-clinical manifestations (Tsuiji and Yamanaka, 2014).

Genetic factors and natural aging processes promote the misfolding and aggregation of neurotoxic proteins (Brown et al., 2005; Anand et al., 2014), which triggers a dysregulated brain inflammatory response (Sevenich, 2018; Solleiro-Villavicencio and Rivas-Arancibia, 2018). This neuroinflammatory activity is associated with a state of chronic oxidative stress (Solleiro-Villavicencio and Rivas-Arancibia, 2018), both known as hallmarks of NDDs (Mittal et al., 2014), which stimulates microglia and astrocytes activation (Owens et al., 2005; Rothhammer et al., 2018; Sevenich, 2018; Solleiro-Villavicencio and Rivas-Arancibia, 2018). At a later stage of neuroinflammation, when the integrity of the blood brain barrier (BBB) starts to be compromised, there is infiltration of peripheral myeloid cells, which in the long term ends in progressive tissue damage (Rothhammer et al., 2018; Solleiro-Villavicencio and Rivas-Arancibia, 2018). It is important to discriminate autoimmune inflammatory disorders of the Central Nervous System (CNS) from other NDDs. In autoimmune disorders of the CNS, such as ALS and MS, there is an early involvement of the adaptive immune system (namely, T- lymphocytes and B-lymphocytes) with a causative role. While in AD and PD, there are innate immune reactions as part of what is meant to be a protective mechanism. However, the exacerbation of perpetuated proinflammatory triggers (Rivas-Arancibia et al., 2010; Jayaraj et al., 2017; Solleiro-Villavicencio and Rivas-Arancibia, 2018), together with aberrant activity of microglia and astrocytes, makes a crucial contribution to neuronal loss and dysfunction that culminates in neurodegeneration (Rothhammer et al., 2018; Solleiro-Villavicencio and Rivas-Arancibia, 2018).

As most multifactorial disorders, the pathophysiology of NDDs presents a complex development, where a combination of genetic, environmental, and behavioral factors contribute with causal roles (Santiago et al., 2017) (Figure 1). Besides the genetic component, there are other factors influencing disease development. Natural aging is recognized as the greatest risk factor (Brown et al., 2005; Anand et al., 2014; Tsuiji and Yamanaka, 2014). Metabolic disorders such as type 2 diabetes mellitus (T2DM) (Anand et al., 2014; Yarchoan and Arnold, 2014; Arnold et al., 2018) and hyperlipidemia (Anand et al., 2014; Arnold et al., 2018), arise as well-known risk factors associated to progression of NDDs. In addition, researchers have revealed essential epigenetic mechanisms that could also be dysregulated in NDDs. However, such neuroepigenetic modifications are dynamic and to some extent reversible, since they are somatically non-heritable (Hwang et al., 2017).

**FIGURE 2:**
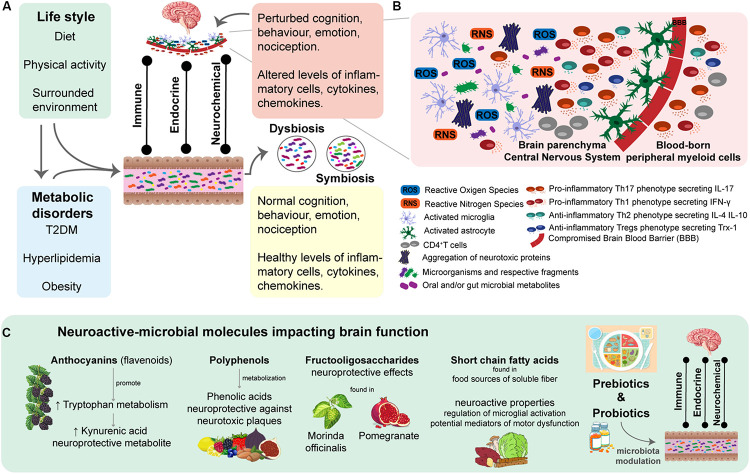
Chronic neuroinflammation associated with disease-specific immune responses is a hallmark of Neurodegenerative diseases (NDDs). Thus, triggers of redox state imbalance and mechanisms promoting a proinflammatory brain response have been the target of therapeutic approaches. **(A)** An individual’s microbiota composition is strongly influenced by life style factors and personal clinical history. At the gut level, the intestinal microbiota plays an essential role in the modulation of the bidirectional gut-brain axis, through immune, endocrine and neurochemical direct and indirect pathways. There is evidence of symbiotic and dysbiotic gut microbiota assuming a preventive or promoter role in development and progression of NDDs, respectively. **(B)** Exacerbated neuroinflammation, due to chronic oxidative stress accompanied by dysregulated inflammatory response, is a Hallmark of NDDs. Increased levels of ROS and RNS stimulates the secretion of proinflammatory molecules (e.g., cytokines and chemokines), which in turn leads to microglia and astrocytes activation. Under these neuro-proinflammatory response, peripheral myeloid cells are recruited to the central nervous system (CNS), which intensifies microglia and astrocyte activation. Such a cascade of mechanisms is capable of perpetuating the proinflammatory response in the brain microenvironment, consequently loosing neuroinflammation regulation in NDDs. There are several mechanisms being studied that promote proinflammatory response, such as mitochondrial dysfunctions, aggregation of neurotoxic plaques, which can be stimulated by the invasion of microorganisms and respective fragments to the CNS, as well as by certain microbial metabolites with brain damaging profile. **(C)** Perturbation of the redox balance state and immune landscape healthy-state of the CNS of specific brain regions underlies disease-specific signatures of the neuroinflammatory microenvironment, which have been targeted in attempted therapeutic approaches. There is a list of studied neuroactive-microbial molecules improving brain function and cognition. These neuroprotective metabolites are microbially metabolized and secreted upon the ingestion of prebiotics. Thus, evidencing the potential of a dietary-based intervention as a complementary therapeutic in NDDs. Moreover, administration of probiotics in order to modulation microbiota has been revealed to be promising due to the role of intestinal microbiota in the bidirectional interactions between the gut and the CNS, as seen through the consumption of Bifidobacterium breve strain A1 by AD mice, which demonstrated a preventive role in cognitive dysfunction.

In the past few years, several studies have focused on the role of microbiota (e.g., nasal, oral and intestinal) and respective metabolites in the promotion, development and prevention of NDDs (Borre et al., 2014; Friedland and Chapman, 2017; Harding et al., 2017; Luan et al., 2017) (Figure 2A). Briefly mentioning, there is evidence of substantial contributions of host microbiota to microglia maturation and function in the CNS (Erny et al., 2015); it has been proposed the microbiota-controlled metabolic inflammation concept in T2DM and obesity (Tilg et al., 2020), interestingly both metabolic disorders have been linked to susceptibilities in cognitive function (Naseer et al., 2014; Agusti et al., 2018; Arnold et al., 2018); identification of CNS-resident and peripheral immune pathways influenced by host microbiota in neurological diseases (Fung et al., 2017); identification of microbial-metabolites related to cognitive functions (Liu et al., 2020), such as learning, memory and decision making processes (Montiel-Castro et al., 2013). All this progress in untangling the contribution of microbiota to host homeostasis and immune responses (Tremaroli and Backhed, 2012; Montiel-Castro et al., 2013; Shoaie et al., 2013; Rosario et al., 2018) emerges the need of a better understanding of the bidirectional communication within the microbiota-gut-brain axis from the NDDs perspective. Besides the increasing body of knowledge of identified dysbiotic microbial species in NDDs, efforts are being made to identify key species playing a functional and mechanistic role in disease. Potential novel therapies may halt or slow down the degeneration processes in the rising burden of NDDs (Wang et al., 2015; Chen et al., 2017; Kobayashi et al., 2017). In this sense, modulation of microbiota composition through microbiota transplantation (Baquero and Nombela, 2012), dietary plans and/or administration of pre-, pro-, and post-biotics has been subject of investigation as potential complementary therapeutic approach in NDDs (Wang et al., 2015; Chen et al., 2017; Kobayashi et al., 2017).

Risk factors contributing to NDDs development are still poorly understood or remain unidentified. Although symptomatically distinct, NDDs share hallmarks and altered mechanisms. By comprehending one disease, it might help to understand perturbations occurring in another disorder. The complexity and exponential rise in numbers of diagnoses of NDDs has driven research into the discovery of novel biomarkers that could provide an earlier diagnosis before the development of notorious neurological symptoms and to identify novel drug targets that can be used in the development of effective treatments (Ma, 2018). Since NDDs are heterogeneous multi-systemic disorders presenting multifactorial etiology, there is a need for a systems-level explanation to abstract all these changes and their interactions to better describe disease mechanisms and direct us to novel therapies. Recently, the role of microbiome in the gut-brain axis has received a notorious increase of attention (Borre et al., 2014; Friedland and Chapman, 2017; Fung et al., 2017; Gerhardt and Mohajeri, 2018). Due to its functional role (Hooper et al., 2012; Tremaroli and Backhed, 2012; Fung et al., 2017; Dalile et al., 2019; Tilg et al., 2020), the microbiome has been considered as a vital organ of the human organism (Mills et al., 2019). There is an emerging interest in comprehend the microbiome’s functional impact through the study of microbe-microbe, host-microbe interactions and in understanding the contribution of these to human physiology in NDDs context (Elizabeth et al., 2017). However, it is difficult to determine the function of colonic (and others) bacterial strains, since microbial behave can alter depending on the presence and abundance of co-existing microbes composing a community (Mills et al., 2019). Systems biology approaches bring the possibility of exploring the cause and effect of host-microbiome interactions as an organ part of the human system (Elizabeth et al., 2017). In this review we will discuss the employment of Systems biology and the integration of multiomics data, with focus on its potential in biomarker discovery and in drug targets identification for development of effective personalized treatments in NDDs based on the human microbiome modulation.

Here, we discuss the role of the microbiome in the gut-brain axis in the context of NDDs. Namely, microbial triggers stimulating neuroinflammation, as well as neuroactive and neuroprotective microbial-molecules improving brain function. We reviewed existing studies revealing evidence of microbial perturbations contributing to PD and AD hallmarks and metagenomics studies identifying disease-signature of shifted microbial abundances. The complexity of the microbial community turns it difficult to understand functional interactions of altered microbes to host metabolic pathways. We expose how Genome-scale metabolic models (GEMs), as the computational platform to integrate multi-omics data, can be implemented to reveal microbe-microbe and host-microbe interactions and therefore, microbial contribution to molecular mechanism underlying disease. We discuss future perspectives integrating microbial and host data based on GEMs, multi ‘omics methods and biological networks to give a comprehensive insight of the role of microbiota to host homeostasis in NDDs. We describe a systemic approach based on such methodologies that would allow us the study and the design of precise and sustainable personalized anti-inflammatory diets in NDDs prevention, as well as a complementary whole-body modeling perspective.

## Neuroinflammation With Chronic Loss of the Brain Balanced Redox State as a Hallmark of NDDs

In normal conditions, there is a balance between the production and activity of reactive oxygen and reactive nitrogen species (ROS and RNS, respectively). Under this healthy balance, immunological responses in the brain aim to preserve brain cells, as well as molecular and biochemical functions (Mittal et al., 2014). However, under pathological conditions threatening the immune system and homeostasis the redox balance is compromised. Thus, certain enzymes (e.g., NOX2, a phagocytic enzyme produced during exposure to pathogens) produce ROS as part of the inflammatory response (Mittal et al., 2014; Solleiro-Villavicencio and Rivas-Arancibia, 2018). Moreover, stimulation by cytokines, growth factors, hyperglycaemia and hyperlipidaemia contribute to such enzymatic activity creating an increased concentration of reactive species (Solleiro-Villavicencio and Rivas-Arancibia, 2018). Additional mechanisms promoting an inflammatory response that subsequently perturbs the brain redox balance are: (1) abnormal mitochondrial dysfunction (Rego and Oliveira, 2003) due to mutations, such as leucine-rich repeat kinase *2(LRRK2)*, *PINK1*, *DJ1*, and α-synuclein associated to PD (Zuo and Motherwell, 2013); (2) brain-infection due to microbial exposure (e.g., by *Candida albicans, Salmonella enterica*) (Stilling and Cryan, 2016) or by proinflammatory microbial-metabolites that migrate and are capable of reaching the brain (Wekerle, 2018).

In a chronic oxidative environment accompanying exacerbated neuroinflammation (Jayaraj et al., 2017; Solleiro-Villavicencio and Rivas-Arancibia, 2018), the anti-oxidant defense systems responsible for the modulation of proinflammatory responses are dysregulated by increased signaling molecules such as ROS (Rivas-Arancibia et al., 2010; Jayaraj et al., 2017; Solleiro-Villavicencio and Rivas-Arancibia, 2018). ROS promote the activation of phosphorylation pathways while inhibiting enzymes responsible for dephosphorylation. Consequently, the dysregulation of cellular transduction signals enhances the secretion of proinflammatory molecules (e.g., cytokines and chemokines) and neoepitopes and stimulates the production of danger-associated molecular patterns (DAMPs) (Solleiro-Villavicencio and Rivas-Arancibia, 2018). Thus, in a state of oxidative stress, increased concentrations of reactive species (e.g., ROS, RNS and free radicals) contribute to the stimulation and activation of microglia and astrocytes. This neuro-proinflammatory response promotes the recruitment of peripheral myeloid cells, which in turn intensifies the activation of microglia and astrocytes, therefore generating a vicious cycle (Gonzalez and Pacheco, 2014; Gonzalez et al., 2014) (Figure 2B). Naturally, with disease progression the phenotype pool of recruited cells into the CNS becomes more diverse, being accompanied by phenotypical changes of microglial cells that lose their quiescent inactive state (Sevenich, 2018; Solleiro-Villavicencio and Rivas-Arancibia, 2018).

Microglia, the innate immune cells of the CNS, and the astrocytes are responsible for the brain immune defense and homeostasis (Sevenich, 2018). Therefore, microglia have been recognized as the surveillance of the CNS (Wieghofer and Prinz, 2016), responsible for the stable immune landscape of the CNS under healthy-state conditions (Wieghofer and Prinz, 2016; Sevenich, 2018). The blood-brain barrier (BBB), essentially generated and regulated by astrocytes (Solleiro-Villavicencio and Rivas-Arancibia, 2018), restricts the access of blood-borne immune and inflammatory cells to the CNS (Sevenich, 2018). A dysregulated activation of the microglia works as a trigger for neuroinflammation, which begins as a neuroprotective mechanism (Hansen et al., 2018). However, under pathological conditions, it is followed by a microglia-induced exacerbated inflammatory response (Sevenich, 2018; Solleiro-Villavicencio and Rivas-Arancibia, 2018). Such mechanisms involve the recruitment of peripheral myeloid cells, responsible for alterations of the healthy-state of the immune landscape and homeostasis of the CNS (Sevenich, 2018). The transmigration of these blood-borne immune and other inflammatory cells to the CNS, due to the loss of BBB integrity, is seen as a critical mediator of the progression of NDDs (Sevenich, 2018; Solleiro-Villavicencio and Rivas-Arancibia, 2018). There is been an emerging interest in approaches focused on the modulation of gut microbiota for the secretion of neuroactive-microbial molecules with impact on brain function with aim to slow down neuroinflammation (Wang et al., 2015; Chen et al., 2017; Kobayashi et al., 2017) (Figure 2C). Later in the review we present a section dedicated to the influence of these complementary dietary-based therapeutic approaches involving the gut-microbiome-brain axis in NDDs.

## The Role of Microbiome in the Bidirectional Communication of the Gut-Brain Axis in NDDs

The human microbiota is a thriving dynamic ecosystem composed by trillions of microbial cells living in symbiosis (Borre et al., 2014), which seems to be host-specific (Donaldson et al., 2016). While the human microbiome consists of the genes and respective gene-products that these microbial cells harbor (Ursell et al., 2012). The microbiome is an emerging field due to the improvement of sequencing techniques (Mardinoglu and Nielsen, 2012) and development of pipelines for downstream analysis of metagenomics data, which have enabled the identification of thousands new microbial species (Qin et al., 2010; Li et al., 2014) living in human phylosymbiosis (Ross et al., 2018). Moreover, the enhancement of other high-throughput techniques and quality of originated data, such as metabolomics and proteomics (Mardinoglu and Nielsen, 2012), has been contributing for a better understanding of host–microbiome interactions, including insights on the essential role of intestinal microbiota in the bidirectional gut-brain axis (Cryan and Dinan, 2012; Borre et al., 2014). Metagenomics, based on the genetic material, will give us insight regards which species are present and at what abundance in a certain context (e.g., the human intestinal microbial ecosystem or the human oral microbiota) (Elizabeth et al., 2017). Analysis of metabolomics data enables the comprehensive profile of small cellular metabolites concentrations. Often, biologic fluids, such as blood and urine, are used as metabolomic samples to have the perception of circulatory and excretory metabolites in health and disease (Mardinoglu et al., 2018a). In the field of human microbiota, metabolomics can additionally be performed using stool samples in order to have information on the microbial-metabolite consumption and production contributing to the human metabolism (Sen and Oresic, 2019). Different ‘omics data will give different biologic information. This biologic insight can be integrated to allow the analysis of molecular mechanisms composing complex networks, which are representative of biological systems (Mardinoglu et al., 2018a; Sen and Oresic, 2019).

**FIGURE 3:**
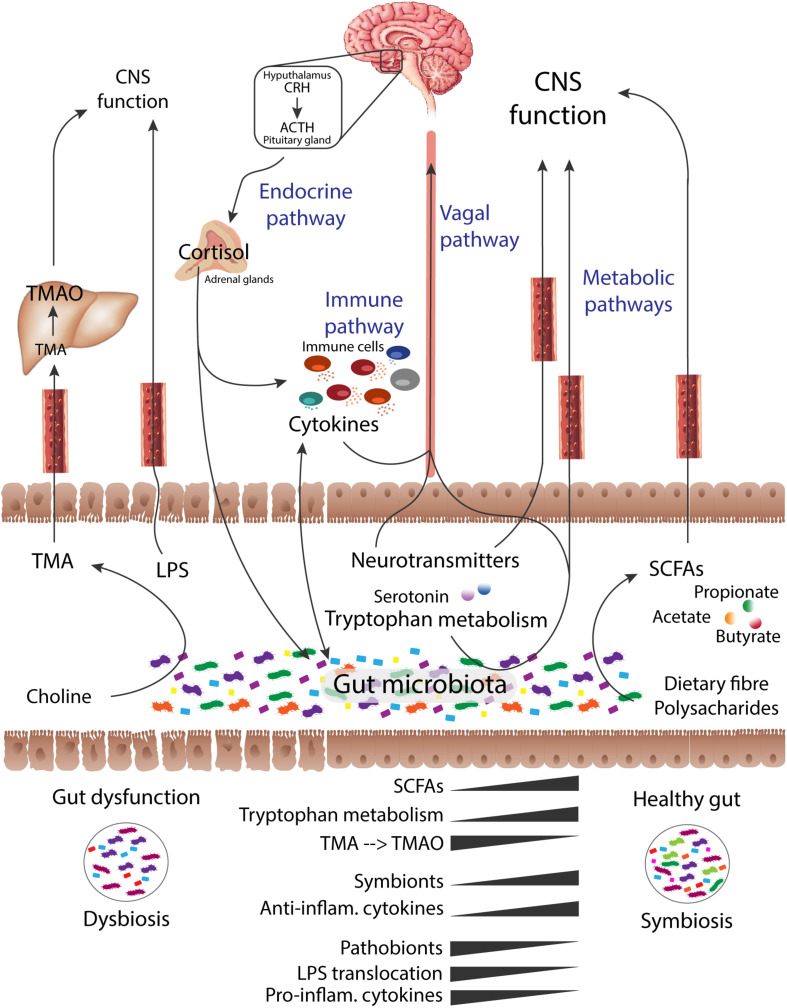
Endocrine, immune, metabolic and vagal direct and indirect pathways for bidirectional communication of the gut-brain axis. Changes in the bacterial abundances and development of gut dysfunctional state (dysbiosis) impacts host and Central Nervous System (CNS) functions, often associated to disease. Consequently, there is a shift in microbial-derived products. Short-chain fatty acids (SCFAs), derived from the microbial digestion of dietary fiber, play a crucial role in the regulation of microglia and brain immune responses. The production of SCFAs, neuroprotective biochemicals essential to host metabolism, is compromised under a state of intestinal dysbiosis, which impacts the CNS function. Increased levels of Trimethylamine *N*-oxide (TMAO), a gut microbial-mediated metabolite, has been linked to aging and cognitive impairment. The microbial metabolism of tryptophan, leading to the production of, for instance, the neurotransmitter serotonin, is also compromised under dysbiotic states. On the other hand, stress at the CNS level can impact intestinal function and promote gut microbial perturbations. Thus, the CNS is capable of recruiting the same mechanisms to modulate the gut microbial composition, such as by the stimulus of cortisol secretion. Cortisol can have an influence on immune cells recruitment and cytokines secretion, as well as on the epithelial barrier permeability. Compromised integrity of the gut epithelial barrier allows the translocation of overgrowth pathobionts and neurotoxic microbial fragments, such as lipopolysaccharides (LPS), which can later reach and cross a compromised blood-brain barrier. The microbes and their secreted metabolites shape the host-immune system and vice-versa. A dysbiotic intestinal microbiota can hijack the host-immune system and modulate the inflammasome signaling. Note: figure was adopted from references (Grenham et al., 2011; Cryan and Dinan, 2012).

Due to its functional involvement in the production of essential bioactive metabolites (Dalile et al., 2019), regulation of immune triggers (Hooper et al., 2012; Fung et al., 2017; Tilg et al., 2020) and host-energy homeostasis (Tremaroli and Backhed, 2012), the human microbiome has been compared to a vital organ of the human organism (Dalile et al., 2019). The intestinal microbiota plays an essential role in the bidirectional gut-brain axis (Cryan and Dinan, 2012; Borre et al., 2014; Stilling and Cryan, 2016), which represents the interplay between the gastrointestinal tract and the central nervous system (Cryan and Dinan, 2012; Borre et al., 2014; Luan et al., 2017) through neural, endocrine and immune mechanisms (Grenham et al., 2011; Cryan and Dinan, 2012; Montiel-Castro et al., 2013; Vogt et al., 2017) (Figure 3). Metabolic processes of the gut microbiota have an influence on the bioavailability of micronutrients in the host-intestinal metabolic pool. These microbial-metabolites produced by colonic bacteria can have a local and/or systemic action when absorbed into the bloodstream (Schroeder and Backhed, 2016). The potential beneficial or toxic impact of these microbial-produced biochemicals will depend on different factors, namely the metabolite concentration and which organ is involved (Tremaroli and Backhed, 2012). In this sense, dysbiosis of the human microbiota is directly linked to alterations in host metabolism. One example is trimethylamine *N*-oxide (TMAO), a gut microbial-mediated metabolite, involved in the increased risk of CVD (Aron-Wisnewsky and Clement, 2016) and also associated to AD (Xu and Wang, 2016).

Metabolic activities of gut microbiota influence host physiology, such as through the degradation of indigestible nutrients. Short-chain fatty acids (SCFAs) are microbial-products from indigestible fiber, which are involved in the human energy metabolism (Tremaroli and Backhed, 2012; Montiel-Castro et al., 2013; Shoaie et al., 2013; Rosario et al., 2018). Thereby, shifts in the microbiota composition leading to dysbiosis may contribute to the development of disorders associated with functional metabolic perturbations (Tremaroli and Backhed, 2012; Donaldson et al., 2016). In the crosstalk between the gut microbiota and the host brain, neuroactive-microbial molecules mediate the regulation of metabolic pathways impacting brain function, such as through the microbial-secretion of SCFAs (Borre et al., 2014; Harding et al., 2017; Luan et al., 2017). In turn, the brain is capable of recruiting the same mechanisms and modulate the gut microbiota composition, such as by the action of cortisol secretion. Cortisol is known by its effect on immune cells and cytokines secretion, as well as by impacting gut barrier and permeability (Cryan and Dinan, 2012). Consequently, under an intestinal dysbiotic environment, the epithelial barrier integrity gets compromised (recognized as the leaky gut). With an inappropriate microbiota-gut-brain crosstalk signaling and intestinal barrier impairment, the translocation of overgrowth pathobionts, microbial fragments or products becomes possible (Borre et al., 2014). Such dysfunctional interaction have been linked to development of neurodegeneration (Montiel-Castro et al., 2013; Borre et al., 2014; Luan et al., 2017), as well as abnormal behavior, cognitive impairment, stress and visceral pain (Cryan and Dinan, 2012; Borre et al., 2014).

Erny et al. (2015) demonstrated that the host microbiota and SCFAs (as their main bacterial fermentative products) play crucial role in the regulation of microglia morphology, maturation and brain immune responses. Studies with germ free (GF) mice revealed impaired innate immune responses with global defects in microglia against infection. The lack of bacteria demonstrated altered microglia proportions with immature phenotype and altered gene profile. Moreover, it was verified malformation of cells and perturbations in cellular networks in microglia from sterile mice. Continuous experiments with temporal eradication of host microbiota and with lower microbiota diversity also revealed severe and defective changes at the microglia level (Erny et al., 2015). Contrarily, the recolonization with increased microbiota diversity demonstrated a partial recover of the microglia phenotype (Erny et al., 2015). In previous studies, SCFAs have demonstrated to be vital for homeostasis of immune cells, namely regulatory T cells, in the colon (Smith et al., 2013). Additional research has shown that microglial defects could be restored by the administration of SCFAs (Erny et al., 2015).

Studies in mice have shown that gut microbial products derived from the dietary essential amino acid tryptophan regulate inflammation in the gut and CNS (Marsland, 2016; Rothhammer et al., 2016). Intestinal microbiota metabolizes tryptophan into a range of indole derivatives, such as indole-3-acetic acid, indoxyl-3-sulfate, indole-3-propionic acid and indole-3-aldehyde. These microbial products are known ligands of the aryl hydrocarbon receptor (AHR). In the colon, activation of AHR of gut-resident T cells and innate lymphoid cells has revealed protective effects against inflammation by stimulating the secretion of interleukin (IL)-22 (Marsland, 2016). At the systemic level, interferon (IFN)-1 signaling limits inflammation in the CNS by activating AHR in astrocytes (Marsland, 2016; Rothhammer et al., 2016). Additionally, Rothhammer et al. (2016) have demonstrated that microbial metabolites derived from tryptophan have an agonist effect on AHR existent in astrocytes. Thus, suggesting that in combination with IFN-1 signaling, the CNS inflammation can be suppressed (Rothhammer et al., 2016). The presented findings give evidence on the influence of the host microbiota in the modulation of the brain innate immune system. This suggests a path to investigate potential treatment of microglia-mediated inflammatory responses in NDDs.

T2DM is a metabolic disease often promoted by obesity-linked insulin resistance (Wellen and Hotamisligil, 2005; Naseer et al., 2014). Additionally, vascular effects observed in obesity have been implied in the development of AD. Thus, there is been a notorious interest in studying simultaneously the role of intestinal microbial changes observed in obesity, T2DM and the further initiation of AD (Naseer et al., 2014; Agusti et al., 2018). A study focused on the gut microbiota modulation in mice models demonstrated that such intervention changed the expression of inflammatory and metabolic genes in the hepatic and intestinal environments, and influenced hormonal secretion and host homeostasis, which promotes improvement in glucose tolerance (Membrez et al., 2008). Other studies have suggested that an obesity-associated microbiota might contribute to alterations of endocrine, neurochemical and inflammatory mechanisms underlying obesity (Agusti et al., 2018), through pathways involved in the bidirectional communication between the gut microbiota and the brain (Cryan and Dinan, 2012).

## Evidence that Microbial Dysbiosis Contributes to the Hallmarks of PD

Parkinson’s disease, a progressive neurological disorder (Chin and Vora, 2014; Tsuiji and Yamanaka, 2014; Ma, 2018) with a multifactorial etiology (Chin and Vora, 2014; Ma, 2018), is the most common NDD compromising motor functions (Brown et al., 2005; Gammon, 2014), affecting 1–2% of people with age over 65 years old (Felice et al., 2016). PD pathophysiology is essentially hallmarked by the degeneration of nigrostriatal dopaminergic neurons in association with the deposition of misfolded α-synuclein in the remaining neurons, culminating in the characterizing motor impairment (Nussbaum and Ellis, 2003; Aarsland et al., 2017). Moreover, as a heterogeneous multi-systemic disorder (Lee and Koh, 2015; Aarsland et al., 2017), besides altered dopaminergic pathways in PD, the serotonergic, noradrenergic and cholinergic systems are additional neurotransmitter circuits pathologically involved in the disease. Thus, leading to a wide-range of non-motor symptoms (NMS), which are commonly reported as precedents of the motor symptoms by several years (Aarsland et al., 2017).

Braak et al. (2006) have shown evidence of phosphorylated aggregates of α-synuclein in neurons encompassing the enteric nervous system (ENS) (Braak et al., 2006), along the entire gastrointestinal (GI) tract (Wakabayashi et al., 1990), and the olfactory bulbs (OB) (Ross et al., 2006). Notably, these are gateways contacting with the external environment (Klingelhoefer and Reichmann, 2015). Theoretically suggesting that the exposure to toxins or pathogens and subsequent cascade of local inflammatory and immune responses triggers detrimental processes associated to PD in the ENS and/or OB. Later, spreading to the central nervous system (CNS), namely to the substantia nigra and higher cortical regions, via vagal nerve and olfactory tract, respectively (Hawkes et al., 2009; Klingelhoefer and Reichmann, 2015). Within the NMS experienced by PD diagnosed individuals, the most commonly reported is GI dysfunction (Savica et al., 2009; Pfeiffer, 2011; Fasano et al., 2015). The increasing body of evidence of an early involvement of the GI tract together with the ENS and hypothetic contribution of environmental factors for the development and progression of PD emerges the interest for a better understanding of interactions happening in the bidirectional gut-brain-axis, known for representing the communication between the GI tract and the CNS (Cryan and Dinan, 2012; Borre et al., 2014; Luan et al., 2017), without disregarding the key role that the gut microbiota might play (Cryan and Dinan, 2012; Borre et al., 2014; Stilling and Cryan, 2016). As well as oral and nasal microbiotas in regard to respective anatomic and functional systems, such as the olfactory tract (Friedland and Chapman, 2017), once there is a dynamic mutualistic host-microbial relationship resulting from millions of years of coevolution (Nicholson and Wilson, 2003; Hooper et al., 2012). These microbial species living in human phylosymbiosis (Ross et al., 2018) are interactively contributing for multiple physiologic connections between the gut, muscle, liver and brain through host–microbiota metabolic, signaling and immune-inflammatory pathways. Thus, the microbiota composition is sensitive to environmental factors, such as diet and medication (Nicholson et al., 2012), and these changes influence the host homeostasis, signaling and immune responses (Hooper et al., 2012; Levy et al., 2017).

### Implementation of Metagenomics to Identify a PD Microbiome-Signature and Potential Metabolic Alterations

Microbiota composition of sigmoid mucosal biopsies and stool samples of PD diagnosed individuals revealed stronger alterations in the intestinal microbiota (Tremaroli and Backhed, 2012; Keshavarzian et al., 2015; Scheperjans et al., 2015). A significant depletion in anti-inflammatory butyrate-producing from the genera *Blautia, Coprococcus*, and *Roseburia* was observed in PD. When comparing the mucosa between PD individuals and controls, PD patients demonstrated significantly increased abundance of putative, pro-inflammatory Proteobacteria of the genus *Ralstonia*, while controls revealed to have the bacteria from the genus Faecalibactirum significantly more abundant. Genes involved in metabolism were significantly lower in the gut microbiome of PD patients, while genes involved in lipopolysaccharides (LPS) biosynthesis and type III bacterial secretion systems were significantly higher in PD individuals. Such perturbations support a proinflammatory dysbiosis contributing to the development and pathogenesis of PD (Keshavarzian et al., 2015). In agreement, it has been reported that other studies looking at bacterial colonic alterations in PD have found decreased abundances of *Faecalibacterium* spp., *Coprococcus* spp., *Blautia* spp., *Prevotella* spp. and of the family Prevotellaceae in individuals diagnosed with PD, and increases of *Lactobacillus, Bifidobacterium, Verrucomicrobiaceae*, and *Akkermansia* (Gerhardt and Mohajeri, 2018). Another study, implementing pyrosequencing of the 16S rRNA gene, analyzed the gut microbiota from stool samples of 72 PD patients and 72 healthy controls. Prevotellaceae was reduced by 77.6% in individuals diagnosed with PD. Furthermore, Enterobacteriaceae abundance was positively correlated with motor impairment, evaluated by severity of postural instability and gait difficulty, suggesting that perturbations of the PD microbiome are related to disease-motor phenotype (Scheperjans et al., 2015). Recently, a metagenomic shotgun analysis was performed in order to infer functional implications of alterations in the microbial and viral gut metagenome of 31 early stage L-DOPA-naive PD individuals, having 28 age-matched controls for comparison. This approach found significantly increased abundances of *Verrucomicrobiaceae* (*Akkermansia muciniphila*) and unclassified Firmicutes, whereas Prevotellaceae (*Prevotella copri*) and Erysipelotrichaceae (*Eubacterium biforme*) abundances were significantly decreased in PD patients. Furthermore, alterations in microbiota involving β-glucuronate and tryptophan metabolisms were verified in PD patients (Tremaroli and Backhed, 2012). Currently, it remains unclear whether perturbations leading to microbial dysbiosis have a causative or consequent role in PD pathophysiology. However, such alterations might contribute for PD progression by stimulating inflammatory cascades underlying gut leakiness. In turn, impairment of the gut barrier allows the translocation of pathogens and toxic bacterial fragments capable of reaching the CNS (Perez-Pardo et al., 2017). Subsequently, such triggers are capable of compromising the BBB integrity and promoting a state of chronic oxidative stress due to e.g., LPS-exposure, which culminates in neuronal loss (Cotillard et al., 2013).

## Evidence that Microbial Dysbiosis Contributes to AD

Alzheimer’s disease, a multifactorial progressive neurologic disease with irreversible loss of neurons (Chin and Vora, 2014; Tsuiji and Yamanaka, 2014; Ma, 2018), is the most common form of dementia (Anand et al., 2014; World Health Organization, 2017). Clinically, AD is characterized by a progressive memory impairment together with perturbations over speech, decision making, judgement, orientation and conscious of the surroundings. Nowadays, a definitive diagnosis can only be made by a *postmortem* autopsy (Nussbaum and Ellis, 2003). Accordingly to clinical manifestations, the hippocampus, essential for learning and memory, is the brain area affected at early stages of AD, with brain lesions spreading with disease progression (Tsuiji and Yamanaka, 2014; Ma, 2018). Besides neuronal loss, AD is pathologically hallmarked by the deposition of extracellular senile plaques, which contain Aβ peptides and neurofibrillary tangles. The later are constituted by hyperphosphorylated microtubular tau protein (Nussbaum and Ellis, 2003), while the Aβ peptides present in the senile plaques of AD individuals are cleavage products of the β-amyloid protein precursor by a group a proteases, namely the γ-, β-, and α-secretases (Hutton et al., 1998). Noticeably, the products from the action of γ-secretase are Aβ peptides with 42 amino acids length (Aβ_42_). The Aβ42 is known for its pathogenic profile in AD once it is capable of forming insoluble toxic fibrils and subsequently it accumulates in the distinctive senile plaques of AD (Esler and Wolfe, 2001).

Evidence of LPS and other gram-negative bacterial fragments co-localizing with amyloid plaques in *postmortem* brain tissue of AD patients (Zhan et al., 2016; Zhao et al., 2017) suggests that microorganisms contribute to the stimulation of neurodegeneration (Stilling and Cryan, 2016). Thus, a dual protective and damaging role of Aβ protein, classified as an anti-microbial peptide, has been suggested due to its neuroprotective functions (Kumar et al., 2016; Stilling and Cryan, 2016). However, as mentioned previously, aggregation of Aβ protein stimulates the cascade of events occurring during a neuronal proinflammatory response. Therefore, this severe amyloidosis culminates in neurodegeneration (Wang et al., 2015; Kumar et al., 2016; Stilling and Cryan, 2016). A recent study has identified the presence of *Porphyromonas gingivalis* (*P. gingivalis*) and respective microbial-products gingipains (major virulence factors that are secreted and transported to the outer bacterial membrane surfaces) in brain samples of AD patients. *P. gingivalis* is a well-known keystone pathogen in chronic periodontitis. Additional *in vivo* and *in vitro* experiments demonstrated that gingipains are neurotoxic and presented detrimental effects on tau protein. In order to target the neurotoxicity promoted by gingipains, small molecule for its inhibition was designed. The inhibition of these toxic proteases in animal models have revealed to reduce the neuroinflammatory response promoted by gingipains by reducing the bacterial load of *P. gingivalis* in the brain, blocked the production of Aβ_1–42_ and rescued neurons in the hippocampus. Currently, the small molecule is under clinical trials with human subjects (Dominy et al., 2019). Such evidence supports the important role and contribution of host oral and gut microbiotas in AD neurodegeneration (Friedland and Chapman, 2017).

### Implementation of Metagenomics to Identify an AD Microbiome-Signature and Potential Metabolic Alterations

A metagenomics study based on bacterial 16S ribosomal RNA (16S rRNA) gene sequencing of 25 AD diagnosed individuals and 25 asymptomatic age- and sex-matched controls was performed in order to identify gut microbiome alterations in AD. Furthermore, the relationship between the microbiome-signature of AD and its pathology was measured based on well-known cerebrospinal fluid (CSF) biomarkers (Vogt et al., 2017). Alterations in abundance of microbial phyla in AD patients, which included decreases in Firmicutes, Actinobacteria, namely *Bifidobacterium* genus and increased Bacteroidetes were verified (Vogt et al., 2017). Decreased Firmicutes in the gut community of T2DM (Larsen et al., 2010; Vogt et al., 2017) and overweight obese (Schwiertz et al., 2010; Vogt et al., 2017) patients has been previously reported in the literature, which again suggests a mechanism by which such metabolic disorders might contribute to AD development and progression (Naseer et al., 2014; Vogt et al., 2017). The intestinal microbiome of AD participants presented decreased microbial richness and diversity, demonstrating a distinct composition compared to controls. Moreover, the levels of differentially abundant genera correlated with CSF biomarkers (namely, Aβ42/Aβ40, p-tau, the ratio of p-tau/Aβ42 and YKL-40) of AD pathology (Vogt et al., 2017).

## Influence of Diet-Based Therapeutic Approaches With Impact on the Gut-Brain Axis in NDDs

The gut microbiota plays a critical role in human digestion, mainly through the breaking down of complex carbohydrates and proteins (Oliphant and Allen-Vercoe, 2019). These microbial-metabolic activities are dependent on the composition of the intestinal microbial community, which has been associated with long-term diets (Wu et al., 2011). Precision microbiomics emerges this way with two approaches: the use of the gut microbiome as a biomarker to predict responsiveness to dietary plans in order to design precision diets; and the modulation of the gut microbiota toward its contribution to optimal health (Mills et al., 2019). The human microbiome in personalized medicine is seen as the key to move from host-genomics to host-microbiomics in order to achieve precise and functional medicine (Shukla et al., 2015).

There are several approaches involving the modulation of gut microbiota in NDDs with aim to slow down neuroinflammation. The intake of prebiotics and probiotics has been shown to improve host cognition due to their potential preventive and therapeutic role in NDDs (Wang et al., 2015; Chen et al., 2017; Kobayashi et al., 2017), including AD and PD. Other approaches are based on the microbial digestion and production of beneficial metabolites for the host-metabolism. Phenolic acids, which are products from microbial metabolization of dietary polyphenols, interfere with the formation of neurotoxic aggregates, revealing them to be neuroprotective (Wang et al., 2015). Other studies have reported neuroprotective effects of prebiotics, such as fructooligosaccharides (obtained from the plant *Morinda officinalis*) (Chen et al., 2017) and pomegranate (Yuan et al., 2016) in AD animal models. Moreover, the probiotic consumption of *Bifidobacterium breve* strain A1 by AD mice demonstrated a preventive role in cognitive dysfunction (Kobayashi et al., 2017). A previous investigation based on a systems biology approach studied underlying pathways between AD and its biomarkers. This study identified the involvement of TMAO, a gut microbial metabolite generated from a meat and fat based diet, in the development of AD (Xu and Wang, 2016). Thus, it demonstrates that the impact of gut microbiota in the progression and maintenance of neurodegeneration may lead to novel interventional approaches based on the identification of harmful and protective bacterial metabolites (Xu and Wang, 2016; Vogt et al., 2017). A complementary diet-based preventive and therapeutic intervention in NDDs appears to be essential, since it plays a crucial role in the modulation of gut microbiota composition (Cryan and Dinan, 2012; Borre et al., 2014; Harding et al., 2017).

## Neural Routes and Host-Specific Microbiota Influence the CNS in NDDs

As the evidence has shown, the interest in studying host-specific microbiota in NDDs goes beyond the role of intestinal microbiome, which involves the autonomic nervous system in particularly the vagus nerve (Friedland and Chapman, 2017). on PD, evidences has shown the deposition of phosphorylated aggregates of α-synuclein in neurons encompassing the ENS (Braak et al., 2006), along the entire GI tract (Wakabayashi et al., 1990) and olfactory bulbs, respectively (Ross et al., 2006). Thus, besides the study of the contribution of the gut microbiota to the disease, there is also an interest in understanding the role of nasal microbiota for the pathophysiology of the disease (Ross et al., 2006), which might spread via the olfactory receptors in the roof of the nose (Friedland and Chapman, 2017). Additionally, the evidence of the presence of *P. gingivalis* and respective microbial-products gingipains in brain samples of AD patients have revealed the contribution of oral microbiota to the development of disease (Dominy et al., 2019). Thus, the trigeminal nerve in the mouth and nasopharynx may work as neural route by which oral and nasal microbial communities influence the CNS (Friedland and Chapman, 2017).

Investigation of the volatile metabolites in sebum samples from the upper back of PD patients has found hippuric acid as a contributor to the distinctive odor of PD patients (Friedland and Chapman, 2017). Previous studies have associated hippuric acid with gut microbiota (Wikoff et al., 2009). Such developments emerge the interest for including the study of skin microbiome in PD (Friedland and Chapman, 2017).

## Systems Biology Approach as an Integrative Platform to Reveal the Role of Microbiome in the Gut-Brain Axis of NDDs

**FIGURE 4:**
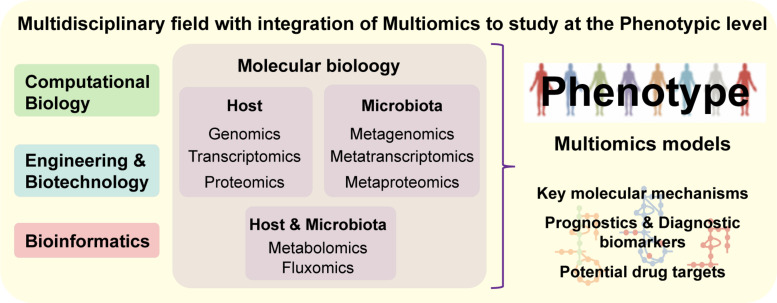
Systems Biology is a multidisciplinary field combining experts from distinct scientific areas and integrating multiomics data, in order to understand complex systems at the phenotypic level. Based on experimental-derived knowledge, systems medicine allows the analysis of molecular mechanisms underlying complex networks representative of biological systems, which makes it an approach with great potential for identification of diagnostic biomarkers and/or drug targets.

**FIGURE 5:**
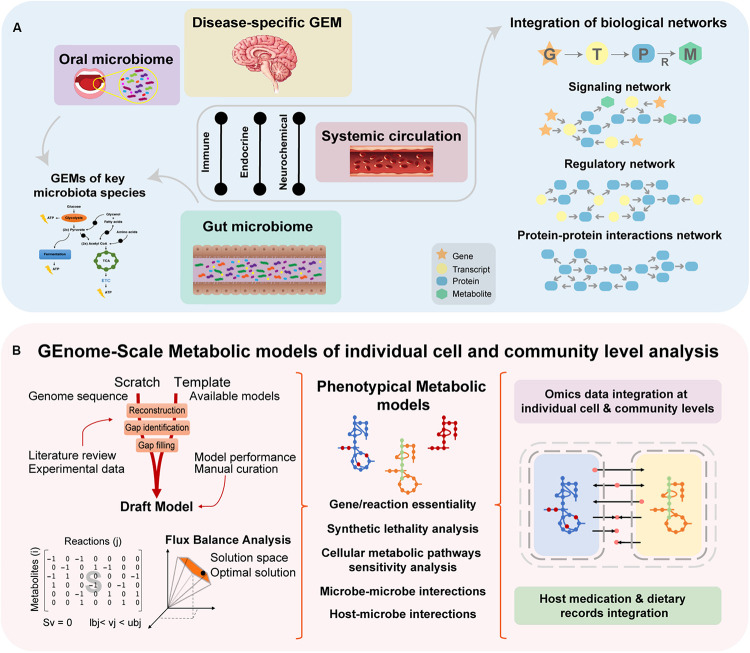
A proper understanding of NDDs complexity requires a holistic approach, since it is challenging to identify key cellular and molecular mechanisms that culminate in such phenotypes. **(A)** Systems biology approaches aim to understand biological interactions occurring within different biologic entities by utilizing mathematical models and network biology representing existing connections between cells and/or tissues. Integrating the data into biological networks, allow to understand interactions between signaling and regulatory pathways occur within the system. **(B)** Reaction-associated enzymes and encoded genes are represented in GEMs (Orth et al., 2010; Zhang and Hua, 2015) with stoichiometric (mass and energy) balance, which enables flux balance analysis (FBA), the study of systemic metabolic responses and analysis of the flow of metabolites through the network (Orth et al., 2010; Mardinoglu and Nielsen, 2012; Zhang and Hua, 2015). Essentiality analysis (EA), based on FBA, works at a single level and allows the identification of essential genes and reactions, the knockout or inhibition of which would interrupt a vital biological function. Complementary, synthetic lethality analysis (SLA) can identify combinations of genes or reactions that when simultaneously knocked out or inhibited can disrupt an essential biological function (Zhang and Hua, 2015).

As a holistic approach, systems biology offers an integrative approach to study the interplay between the different biologic systems as part of a whole (e.g., cells, tissues, organs) (Mardinoglu and Nielsen, 2012). These integrative systemic approaches allow us to move toward a more precise, personalized and translational medicine (Mardinoglu et al., 2018a). Systems biology is a multidisciplinary field that combines complementary scientific expertise such as, cellular and molecular biology, bioinformatics, bioengineering and computational science, in order to understand complex systemic interactions at the phenotypic level (Figure 4) (Auffray et al., 2009). There are two approaches in systems biology: bottom-up, which integrates all known components and interactions to model a system; top–down, where the whole system is decomposed into parts and interactions. Both approaches can be complementary, once the final aim is to know all the components and interactions comprising an intact physiologic system (Mardinoglu and Nielsen, 2012). Large ‘omics datasets (e.g., genomics, transcriptomics, proteomics, metabolomics, fluxomics, and metagenomics) are integrated into computational models and biologic networks (Figure 5A) to give insight and comprehend the dynamic behavior of the system (Mardinoglu et al., 2018a).

The main aim of the systems biology approach is to understand the complexity of interactions by creating biological networks and utilizing modeling. When studying the human microbiome, this would, for example, represent the interactions between the microbial cells and the surrounding ecosystem (e.g., human gut). The main biological network applied in systems biology are GEMs (Mardinoglu et al., 2018a). Integration of experimentally derived data with GEMs has elucidated the molecular mechanisms that occur within complex biological networks (Orth et al., 2010; Shoaie et al., 2013; Zhang and Hua, 2015; Rosario et al., 2018). These approaches allow the identification and understanding of vital interconnected metabolic processes underlying a phenotype of interest (Orth et al., 2010; Mardinoglu and Nielsen, 2012). GEMs have been implemented in previous studies to understand mechanisms underlying insulin resistance (Varemo et al., 2015; Zhang and Hua, 2015), non-alcoholic fat liver disease (Mardinoglu et al., 2014; Zhang and Hua, 2015), interactions between the host and microbiota as well as the effect of the microbiome composition on host metabolism (Shoaie et al., 2013; Ji and Nielsen, 2015; Mardinoglu et al., 2015; Zhang and Hua, 2015). There is a section in this review dedicated to the implementation of GEMs with integration of ‘omics data in the reconstruction of brain and disease-specific models, as well as bacterial metabolic models.

Other widely applied biologic networks in systems biology are gene regulatory networks (GRNs), protein-protein interaction networks (PPINs), gene co-expression networks (GCNs), and signaling networks (SNs) (Mardinoglu et al., 2018a). Interactions happening between transcription factors and genes are represented in GRNs. Based on transcriptional regulatory network principles, GRNs highlight the control of spatiotemporal gene expression (Gerstein et al., 2012). GCNs are another successfully implemented approach to understand associations between gene expression which are translated into functional connections (Lee et al., 2017). Lastly, SNs resemble signaling pathways happening between cellular receptors and different cellular organelles. SNs methods help to determine how information is processed by the cell and to comprehend cellular relationships (Jordan et al., 2000; Azeloglu and Iyengar, 2019). Moreover, dynamical modeling of SNs enables the understand of the impact of a stimuli in a system over time. Thus, based on the computational modeling of cell-signaling networks, it is possible to ignore small or transient signals, while amplifying the focus on cellular signals stimulating physiological functions associated to cell states of interest (Azeloglu and Iyengar, 2019).

PPIs networks provide insight into the functional organization of pathway-components (Bossi and Lehner, 2009). Disruptions of normal patterns of PPIs and multi-protein complexes, which perform key roles in cellular mechanisms, could be the cause or indication of pathology (Kuzmanov and Emili, 2013). PPIs are physical associations occurring between proteins, conferring biological functions at the cell and tissue level (Bossi and Lehner, 2009). Systematic large-scale mapping of physical interactions to investigate mechanisms underlying a disease state have been greatly supported by developments in mass-spectrometry (MS)-based proteomics (Kuzmanov and Emili, 2013). A molecular level landscape of diet-gut microbiome interactions based on PPIs has allowed to demonstrate the impact of phytochemicals on changes in functionality and activity of the gut microbiota (Ni et al., 2015). Food-disease associations were mapped into a network as well as gut microbiota-specific protein target of the food phytochemicals. Based on centrality, a network measurement, was possible to identify the most “vulnerable” bacterial proteins. This mechanistic understanding of associations between the microbial genes/proteins and dietary molecules allow the identification of potential targets belonging to specific bacteria impacting the human health (Ni et al., 2015). Thus, PPIs appear to have potential methodologic application in the field of microbiota modulation based on dietary approaches. As we have mentioned previously, it is an emerging therapeutic field of interest in the context of NDDs. Another interesting application of PPIs in NDDs is to understand interactions between the microbiota and host immune system (Jia et al., 2014). Module-based functional pathway enrichment analysis of PPIs was designed to understand the effects of intestinal microbiota depletion in mice. The results have shown the depletion of gut microbiota affects cellular metabolism, oxidation reduction and neuropeptide signaling pathways. Additionally, such approach allows the identification of candidate genes/proteins and processes related to the interactions between the gut microbiota and the intestinal tract (Jia et al., 2014). Given the hallmarking role of systemic and neuroinflammation in NDDs, PPIs seem as a suitable methodology to be applied to disentangling the role of microbiome in inflammation in NDDs.

Systems biology approaches have been successfully employed in the area of NDDs in an attempt to identify biomarkers and drug targets (Goni et al., 2008; Kuzmanov and Emili, 2013; Su et al., 2018). The modeling of biological networks works as functional tools for the exploration and integration of multiomics data (Mardinoglu and Nielsen, 2012; Mardinoglu et al., 2018a). These holistic and integrative methodologies enable a comprehensive analysis of biological functions, which allows the identification of shifts between healthy and disease conditions (Mardinoglu et al., 2018a). Therefore, such integrative tools not only enable the simulation of brain functions, but also to look closer at the crosstalk happening within the gut-brain axis, with exclusive attention to the crucial role of the microbiota to host metabolism (Nicholson and Wilson, 2003).

## Integration of ‘Omics Data in Order to Reconstruct Brain, Disease and Dysbiosis-Specific Metabolic Models

One approach to study metabolic pathways underlying NDDs is based on the reconstruction of context-specific metabolic models (Sertbas and Ulgen, 2018). GEMs have enabled the identification of key metabolic pathways within a cell, tissue or organism (Zhang and Hua, 2015; Sertbas and Ulgen, 2018) by connecting genes, proteins and metabolites into a functional metabolic model (Mardinoglu and Nielsen, 2012; Agren et al., 2013; Shoaie et al., 2013; Rosario et al., 2018). The reconstruction and performance of these metabolic models are conditioned by the quantity and quality of the integrated data with respect to genetics, physiology and metabolism of the target organism (Mardinoglu and Nielsen, 2012; Agren et al., 2013; Sertbas and Ulgen, 2018) (Figure 5B). Cakir et al. (2007) started the development of a stoichiometric model of the healthy brain-specific metabolic network, which comprised pathways such as the central carbon, amino acid and lipid metabolisms, ROS detoxification and well-known coupling reactions between astrocytes and neurons. Initially, the brain-specific reconstruction integrated 217 reactions and 216 metabolites, while simultaneously implementing a basal physiologic and hypoxic behavior characteristic of the brain cells (Cakir et al., 2007). Further development of this work led to the curation and improvement of the brain-specific GEM by Sertbas et al. (2014). In this improvement, the number of involved metabolic reactions expanded (to 630 reactions and 570 genes), therefore increasing the representation of the brain metabolic pathways (Sertbas et al., 2014).

Disease-specific GEMs have been already developed for a broad range of common NDDs based on ‘omics data (e.g., transcriptomics) (Sertbas and Ulgen, 2018). As example, in a systematic effort transcriptomics data from AD, PD, ALS, MS, HD and schizophrenia have been used from Gene Expression Omnibus, a functional genomics database repository in order to reconstruct disease-specific metabolic models (Sertbas et al., 2014). Model predictions regarding perturbation of metabolic pathways (e.g., oxidative stress, energy including the TCA cycle, amino acid and lipid metabolisms) and transcription factors of regulation (e.g., *USF1*, *SP1*, and *FOX* families) were in agreement with what is reported in the literature of the modeled diseases (Sertbas et al., 2014).

A tissue-based map of the human proteome, by using 24,028 antibodies for protein (antigen) targeting based on immunohistochemistry, allowed the reconstruction of a brain tissue-specific model, specifically on the cerebral cortex region. Here, to understand the spatial human proteome and to validate the proteomic output, the authors performed RNA-Seq from 32 human tissues, turning this approach into an integrative omics application. In this study, the brain was revealed to be the tissue with the second largest number of tissue-enriched genes, with the specific-model comprising 5788 metabolic reactions and 2571 genes, available at the Human Metabolic Atlas (Uhlen et al., 2015). Such computationally reconstructed predictive models give a global representation of the human tissue metabolic networks (Thiele et al., 2013).

Lewis et al. (2010) develop a large-scale metabolic model of interactions between astrocytes and neurons focused on AD. The multicellular metabolic model of the brain has been reconstructed by integrating gene expression and proteomics data. In order to enable the study of multicellular metabolic processes happening in such microenvironment, transfer of metabolites between cells via the interstitial fluid were added as transport reactions. The extensive analysis allowed to identify genes, metabolic pathways and cholinergic neurotransmission involved in AD. The predictions demonstrated that brain regions metabolically affected in AD, namely the hippocampus, the middle temporal gyrus and posterior cingulate cortex, revealed a significant suppression of central metabolic pathways (e.g., glycolysis and the TCA cycle), while regions metabolically less affected by the disease demonstrated no significant suppression. Moreover, *in silico* predictions, in agreement with experimental data, demonstrated a decreased activity of AKGDm in glutamatergic and cholinergic neurons involved in AD, while not in GABAergic neurons, which reflects cell-type and effects of disease in brain regions (Lewis et al., 2010). Thus, multicellular and modeling of metabolic processes occur within cells, between cells and host-microbiota or host–pathogen interactions provide great insight regarding physiology once it is capable of predicting cellular functions and responses to medical interventions.

As previously mentioned, GEMs have been implemented to better understand interactions between the gut-microbiota and the host metabolism (Shoaie et al., 2013; Ji and Nielsen, 2015; Mardinoglu et al., 2015; Zhang and Hua, 2015). Besides systems biology approaches that allowed the analysis of single organism contribution and diet influence to host homeostasis (Shoaie et al., 2013; Mardinoglu et al., 2015; Kovatcheva-Datchary et al., 2019), there have been efforts in the field to develop approaches enabling the modeling of microbial communities (Zengler and Palsson, 2012; Zomorrodi and Maranas, 2012; Manor et al., 2014; Marsland, 2016). Therefore, based on multi-species microbial systems, it is possible to study the trade-offs and relationships (e.g., mutualism, synergism, commensalism, parasitism or competition) between bacteria within a community of interest (Zomorrodi and Maranas, 2012), such as the metabolic-driven analysis of the gut microbiota in NDDs patients. Thus, it is possible to study potential effects of microbial dysbiosis in disease development and progression, as well as the impact of diet on such community. Additionally understanding the diet-microbe and host-microbe interactions, it allows to investigate the interactions within a specific microbial communities as representative of dysbiosis signatures in diseases (Marsland, 2016).

## From Simulation to the Design of Personalized Anti-Inflammatory Diets for Prevention of NDDs; Future Perspective

Aging is the greatest risk factor for the development of NDDs (Brown et al., 2005; Anand et al., 2014; Tsuiji and Yamanaka, 2014). Inflammageing is a neologism reflecting the concept that the natural aging process is accompanied by a global reduction in the capacity to cope with various stressors with a concomitant progressive susceptibility to inflammation with augmented levels of pro-inflammatory markers (Franceschi et al., 2000). Systemic chronic inflammation (SCI) underlies a series of life-style associated disorders, including NDDs and respective comorbidities, such as T2DM. Therefore, there is an emerging interest in identifying potential strategies for early diagnosis, treatment and prevention of SCI in the context of NDDs (Furman et al., 2019). Studies have investigated the role of human microbiome plays in triggering chronic inflammation (Cryan and Dinan, 2012; Fung et al., 2017). The microbiota produces thousands of small molecules and metabolites with systemic impact on the host physiology, which open doors to explore microbial and metabolite-based immune-therapeutics (Skelly et al., 2019). Metagenomics studies will allow to identify perturbations over the homeostatic microbiota composition in health and disease. In-depth functional annotation has the potential to identify effector microorganisms that causally affect the host phenotype and that might contribute to disease aggravation. GEMs, together with ‘omics integration, enable the understanding of effects of microbial-derived molecules and metabolites and their contribution to host physiology (Elizabeth et al., 2017).

Systems biology approaches have shown that it is possible to predict the outcome of personalized designed dietary plans, as well as individual’s dietary records (Shoaie et al., 2015; Mardinoglu et al., 2018b). As previously mentioned, diet strongly modulates the microbiota composition (Nicholson et al., 2012). The study of the nutritional impact of diet-based complementary therapies for NDDs is possible using GEMs representing the gut microbiota community. Such approach has the potential toward a more precise personalized medicine in the field of NDDs. Besides the interest in foods improving the neuroinflammation progressing in an aging brain, the research field is interested in essential nutrients contributing to the maintenance of brain health and function, such as cognition and learning. A nutritional environment rich in antioxidants and anti-inflammatory properties seems of high relevance in prevention and complementary treatment of NDDs (Whalley et al., 2004). The gut microbiota is involved in the bioavailability of some of these neuroprotective sources (Wang et al., 2015; Yuan et al., 2016; Chen et al., 2017). Phenolic acids (Wang et al., 2015), flavonoids (Vafeiadou et al., 2007; Vauzour et al., 2008; Spencer et al., 2012), omega-3 fatty acid (FA) (Derbyshire, 2018), B vitamins (Kennedy, 2016), and curcumin (Ray and Lahiri, 2009) are examples of the diverse array of interesting brain nutritional bioactive molecules. We live in an overpopulated world with an aging population undergoing a period of climate change with extreme impact on food availability and sustainable production. Besides health benefits, nutritional sources must come from a sustainable and affordable origins (Willett et al., 2019). As example, the intake of omega-3 FA has been focus of research as a preventive approach supporting brain health across the lifespan (Derbyshire, 2018). However, there is a concern in reaching the recommended intake of omega-3 FA from cold water fish supply. Efforts are being made to identify sustainable alternative options of omega-3 FA that would be biologically and cost effective (Deckelbaum and Torrejon, 2012; Nichols et al., 2014). Alternative sources of omega-3 FA under study are flaxseeds, echium, walnuts, and algal oil (Lane et al., 2014). GEMs enable the prediction of different diet effects (Shoaie et al., 2015; Mardinoglu et al., 2018b). Such approach allows the study of different food sources with the same potential systemic effect, for instance neuroprotection. This systemic approach, based on the study of the gut microbiota changes with diet and derived microbial-products, has the potential to design precise and personalized anti-inflammatory diets to be implemented in preventive and functional therapeutic approaches in NDDs. As well as the validated-prediction (e.g., metabolomics integration) of diets with potential harmful effects to the human homeostasis (e.g., metabolic impact of a diet rich in highly processed foods).

In the NDDs area, there are still several challenges to identify key cellular and molecular mechanisms in perturbed metabolic pathways that result the disease phenotypes (Orth et al., 2010; Mardinoglu and Nielsen, 2012). Moreover, there is an increasing interest in comprehend the bidirectional cross-talking between the microbiota and the gut-brain axis (Fung et al., 2017). A systemic approach capable of integrating the microbiome and interections with the immune and nervous systems in NDDs context is required. We purpose a whole body perspective based on the integration of microbial, host-organ-specific GEMs (e.g., brain models and brain-disease models), multiomics data and dietary records/plans. In this way, the functional role and contribution of microbiota (e.g., intestinal, oral, nasal) to the pathophysiology of NDDs can be accessed. Such approach has the potential to investigate the effect of neuroactive-microbial molecules regulating metabolic pathways influencing the brain function. Complementary, ‘omics data can be integrated into other biological networks (e.g., signaling networks or protein-protein interactions networks) to provide systemic insight regarding interactions of interest. Thus, a holistic approach for better understand multisystemic interactions and perturbations of NDDs focused on the role of the microbiome is possible, which might potentially reveal novel effective solutions.

## Author Contributions

DR, SS, and AM conceived and presented the review idea. DR performed the literature search, developed the theory, wrote the manuscript, and originated the figures. All the authors have revised and contributed to the final manuscript.

## Conflict of Interest

The authors declare that the research was conducted in the absence of any commercial or financial relationships that could be construed as a potential conflict of interest.
